# Intrinsically Conducting Polymer Composites as Active Masses in Supercapacitors

**DOI:** 10.3390/polym15030730

**Published:** 2023-01-31

**Authors:** Md. Ikram Ul Hoque, Rudolf Holze

**Affiliations:** 1 Discipline of Chemistry, The University of Newcastle, University Drive, Callaghan, NSW 2308, Australia; 2 Australian Institute for Bioengineering and Nanotechnology (AIBN), The University of Queensland, Brisbane, QLD 4072, Australia; 3 Department of Electrochemistry, Institute of Chemistry, Saint Petersburg State University, 7/9 Universitetskaya nab., 199034 St. Petersburg, Russia; 4 Institut für Chemie, Chemnitz University of Technology, D-09107 Chemnitz, Germany; 5 State Key Laboratory of Materials-Oriented Chemical Engineering, School of Energy Science and Engineering, Nanjing Tech University, Nanjing 211816, China

**Keywords:** supercapacitor, capacitive storage, intrinsically conducting polymer, pseudocapacitive

## Abstract

Intrinsically conducting polymers ICPs can be combined with further electrochemically active materials into composites for use as active masses in supercapacitor electrodes. Typical examples are inspected with particular attention to the various roles played by the constituents of the composites and to conceivable synergistic effects. Stability of composite electrode materials, as an essential property for practical application, is addressed, taking into account the observed causes and effects of materials degradation.

## 1. Introduction

Every combination of an active mass designed for use in a supercapacitor electrode with a second component when preparing an electrode may be called a “composite” or a “blend”, with the latter term used only very infrequently in this field (e.g., [1]); the term “hybrid” (material), suggesting specific interactions between the two constituents, should only be used in cases of verified interaction effects only (for an introduction and overview, see [2]). The approach suggested in [3] to use the adjective hybrid in materials with both organic and inorganic constituents with and without interactions appears not to be very helpful. Presumably, a hybrid is better used for materials for which interactions between constituents result in further capabilities of the material beyond simple addition. In an even wider and presumably somewhat misleading interpretation of the term, even the use of a graphene paper as current collector coated with an ICP has been called a composite [4]. Given that for practically all battery and supercapacitor electrode materials the active mass is combined with a binder resulting in better coherence between the active mass particles and adherence to the current collector, and with a conducting additive (carbonaceous material) for enhanced electronic conductivity, these materials may already be called composites. Apparently, this terminology is not generally applied; instead, the term “composite” is frequently and preferably applied only to combinations of two or more active materials. A typical example is the combination of an intrinsically conducting polymer ICP like polyaniline PANI, polypyrrole PPy, or poly-3,4-ethylenedioxythiophene/polystyrene sulfonate PEDOT/PSS (for more see figure below) with a metal chalcogenide like MnO_2_. Different from the obviously non-standard classification presented in [5] in which metal oxides are not included in the class of chalcogenides (this overview contains further gross misrepresentations regarding metal chalcogenides), in the present report, metal compounds with all group 16 elements are considered. Both constituents, the ICP and the metal oxide, are redox-active and thus contribute to the Faradaic charge storage. In addition, both components have an electrochemically active surface in contact with the electrolyte solution where electrochemical processes happen; this interfacial capacity (double layer capacitance) also contributes through capacitive charge storage. As already pointed out long ago by Conway when coining the term “pseudocapacitive”, the latter truly capacitive contributions are small by comparison, and they may amount to a few percent of the redox storage capability [6].

The other “active mass” beyond ICP and metal chalcogenides is high surface-area carbon in its numerous forms. With these carbonaceous materials charge storage proceeds only in a capacitive way, the small amount–if any–of surface redox groups is mostly of minor importance only. Although there are (practically) no redox processes going on, this material is also sometimes called active, and it certainly is so in the present context. Indeed, it is mixed with exactly the same additional ingredients already mentioned above, and thus, it does not come as a surprise that combinations of ICPs with carbonaceous materials are also frequently encountered as composites in the literature. Again, the question for the roles played by the two composite constituents should be asked in order to garner a better understanding of the operation of these materials and of their advantages and drawbacks. The twin roles played by carbonaceous materials may possibly provide cause for confusion when including a given material in this overview. In order to minimize this risk, preferably reports explicitly identifying this carbonaceous material as a composite constituent are included.

In a review on composite materials of ICPs and metal chalcogenides provided elsewhere [7], a substantial number of materials have been inspected. This number, as well as the number of reports dealing with combinations of carbonaceous materials and explicitly named ICP composites, has grown further in the meantime (for an update, see [8]). The present report will not repeat and extend the former review [7] or other earlier overviews and reviews covering parts of the field inspected below [9], neither will it attempt to cover every reported composite of an ICP with a carbonaceous material or any other second constituent. It will instead focus on the salient aspects and general questions in an attempt to provide an overview.

Further combinations of materials with ICPs and thus more composites have been studied: combinations of ICPs with metals (see also [7]) and with electrochemically inert but structure-forming materials have been examined. The number of examples is small, and relevant aspects are collected in a further section.

The number of possible applications of flexible and/or stretchable supercapacitors requiring corresponding electrodes and materials has stimulated research in respective materials; for examples and overviews, see [10,11,12,13,14,15,16]. More general reviews and overviews on supercapacitors, their materials, and their applications are available—see, e.g., [3,6,17,18,19,20,21,22,23,24,25,26,27,28,29,30,31,32,33,34,35,36]—and the increase in energy density at the device level is addressed in passing in many of these publications and has been highlighted with respect to practically relevant results in [37]. The latter aspect combined with the advantages of asymmetric and hybrid approaches has been discussed [38]. A general aspect of electrode material investigations and materials application is the amount of material (active mass loading) on an electrode (i.e., the thickness in a rough approximation). Very thin films generally show the highest specific charge storage values because of close-to-optimum mass utilization [39,40]. In case of composite materials, this aspect has been discussed with respect to electrodes with high mass loadings [41]. As is expected and fairly obvious, 3D-structures and morphologies with suitable porosities are highlighted. With ICP-containing composite materials, the participating dopant (counterion) needed in practically all applications for charge compensation of the ICP in its oxidized form have been addressed because of the possibly structure-forming or -inducing capabilities of such anions.

## 2. Fundamentals

An electrode in a supercapacitor is composed of at least two components: an active material and a current collector frequently also acting as mechanical support. As many materials, even carbon-based ones as used in electrochemical double-layer supercapacitors EDLC, lack sufficient electronic conductance, which is an essential prerequisite for application in a high-current supercapacitor, highly conductive carbon-based materials like carbon black or acetylene black barely contributing to the storage process and being otherwise inert are added. Frequently a binder is added in order to provide the necessary adhesion between the various components. The latter two constituents can make up to 30 wt.% or more without contributing to charge storage; accordingly, they will negatively affect the performance data (available charge density). In the case of the materials discussed here, the situation is the same, and surprisingly, attempts to optimize the actual composition and minimize the “dead weight” have rarely been reported.

Redox-active materials have been studied as active masses or at least as constituents in supercapacitor electrodes since early reports about their electrochemical behavior appearing like (“pseudo”, Greek) a capacitive one, as indicated with the term “pseudocapacitive” by Gileadi and Conway [42] and later extended in [6]. The most frequently studied materials showing suitable electrochemical behaviors are metal chalcogenides, especially those formed from mixed metal (for earlier reviews, see [43,44,45,46,47,48,49,50]; for an update, see [51]) and ICPs. For overviews on ICPs and their applications in electrochemical energy technology, including supercapacitors, see [52,53,54,55,56,57]; for selected ICPs, see e.g.,; for PANI, see [1,58,59]; for PPy, see [60]. Electrodes prepared with only a single material have shown significant drawbacks, such as insufficient electronic conductivity, excessive shape change during cycling, and lack of stability. Thus, early composites aimed at improved performance obtained by combining matching properties of the individual constituents [7]. As another option, suitably structured materials have frequently been studied.

In most of the reports suggesting composite materials as active masses for supercapacitor electrodes, neither the intended function of the sometimes numerous constituents nor reasons for the selection of the employed materials are given. These functions can be conceived for

– ICP
  - material for charge storage
  - mechanical binder
  - mechanical stabilizer compensating volume changes
  - electronic conductance enhancer
  - active mass dissolution inhibitor
– second constituent
  - material for charge storage
  - template for structuring ICP morphology
  - structural/mechanical support for ICP

**Figure 1:**
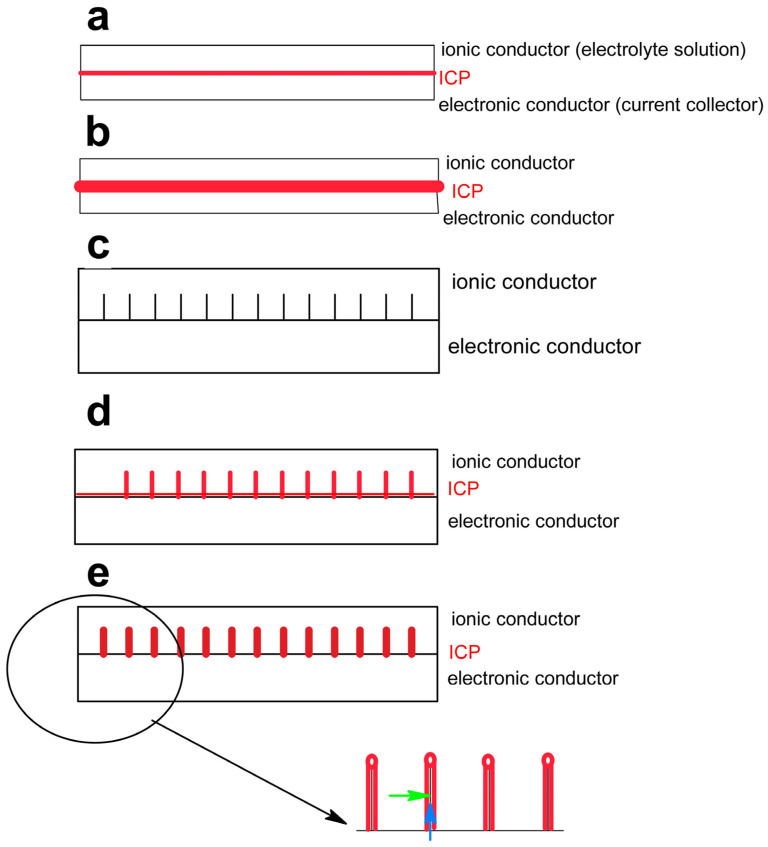
Schematic supercapacitor electrode architectures (bottom right: black: electronically conducting support; red: active mass with ion (→) and electron (→) pathways, further explanations see text).

Some of these tasks are related to the structure, architecture, texture, and morphology of an electrode. They can be found in more-or-less homogeneous combinations of both constituents as well as in combinations where the ICP has been applied as a “top coat”. Some of the various tasks can be depicted in a simplified and highly idealized schematic design of electrode/electrolyte solution architectures (Figure 1). In most experiments simple thin coatings of the active mass—whether it is a single component (in Figure 1 an ICP) or a composite—is studied: Figure 1a. This architecture enables good material utilization and large current capability since the short ways ions and electrons have to move. This design is only of limited practical use, because the mass loading per surface area of an electrode and the final conceivable device will be impractically small. Such thin coatings are instead of scientific interest because the present very high mass utilization and calculated charge capacities [40]. Bigger storage capability can be obtained by increasing thickness of the ER coating, as is shown in Figure 1b. The increased distances that electrons and ions have to travel in turn increase the internal Ohmic resistance (ESR in the complete device); this can decrease or at least limit the current/rate capability. An increased interfacial area is needed, as is shown in Figure 1c. The active material is coated (conformally) onto the three-dimensional support in Figure 1d. The way the pathway’s ions and electrons now have to move will determine the current capabilities, as is shown in Figure 1e and the insert.

In a slightly different way, various architectures have been categorized into anchored, wrapped, and sandwich models [61]. Examples from the various categories demonstrate the particular task of the constituents when, e.g., anchoring a polymer chain on a functionalized carbon nanotube CNT surface. A broader consideration of 3D-electrode architectures, including synthetic approaches, has been reported in [62]. The same applies to the nanostructuring of electrode materials [22,63,64,65] and nanoporous materials [66]. The advantages of 1D-nanostructures with ICPs and their composites when used for supercapacitor applications have been reviewed elsewhere [67]. Materials aspects focused on nanostructured materials, with specific attention to asymmetric supercapacitors, have been discussed in [68,69].

Some tasks are not evident or explicitly addressed in Figure 1. Among them is the capability of an ICP to embed a second active ingredient, like a polyoxometalate, present in small particles. They are somewhat soluble in the electrolyte solution; this will result in capacity losses and subsequently performance deterioration. The ICP restrains this dissolution; for an overview of composites with polyoxometalates demonstrating the combined activities of both constituents, see [70]. Particularly noteworthy for this combination are the linear curves in galvanostatic charge/discharge measurements (GCD), suggesting that strong electronic interactions between the ingredients result in shifts of individual redox potentials yielding current waves in CVs as discussed in [71].

For all of the electrode materials, both single-component and composite, these pathways, together with an adequately extended interfacial area and an appropriate porosity, must be balanced in order to achieve high charge storage and rate/current capability. As a typical example of the importance of the structural effect for electrode performance, the effects of interconnected pores in hierarchically porous carbons from synthetic polymer and biomass sources has been reviewed [72], for further examples and advantageous applications in composites, see [73]. Dedicated current collector material, structured ICP, and/or metal oxide are sometimes difficult to separate clearly with respect to their function. The many explicitly described or at least indicated architectures in the reviewed research publications are briefly sketched in order to illustrate the electrode architecture derived from the publications.

Whether the rational approach discussed in this section qualifies as a rational design may be a more linguistic question; the title of [74] leaves the authors’ understanding of “rational design” in the otherwise hard-to-digest contribution open. At least the term is mentioned only in the title. Composites with ICPs are apparently of minor importance to the authors.

## 3. The Materials

**Figure 2:**
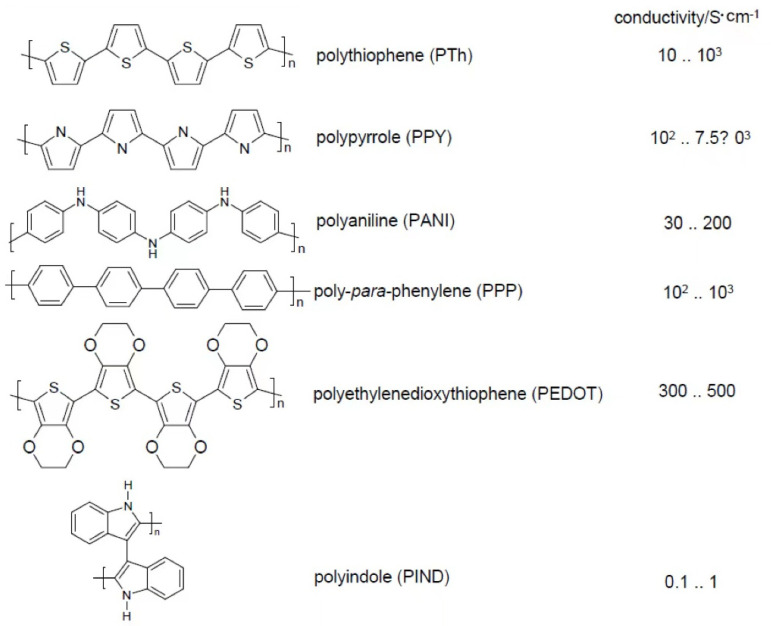
Examples of intrinsically conducting polymers and their monomers (conductivity data presumably—although practically nowhere clearly stated—of the oxidized state of the ICP based on literature sources [most of them secondary without providing original sources of data] [77,78,79,80,81,82,83,84,85,86,87,88]. In the neutral [not to be confused with the reduced] state, the polymers are insulators or semiconductors with conductivities around 10^−5^ to 10^−10^ S·cm^−1^ [89]).

Most of the composite materials covered in this review contain carbonaceous materials or chalcogenides with one metal, like MnO_2_, or with two metals, like NiCo_2_O_4_, as the inorganic constituent. The most-frequently investigated ICPs are PANI, PPy, and PEDOT; PTh and PIND (Figure 2) have rarely been studied. In a review of the composite materials of PTh and its derivatives, not a single application38 in a supercapacitor is mentioned [75]. Considering conjugated polymers and conducting polymers as synonyms, as is the case in [76], appears to be misleading: conjugation is a requirement for conduction, but not all conjugated polymers are conducting. 

Substituted monomers and the corresponding polymers and composites have rarely been studied; in a typical example [1], their application is claimed, but only very few rather distant examples showing no evidence of improved performance are provided. The solubility of some of the latter ICPs may simplify the handling and processing for commercial applications, and consequently, price considerations may turn out to be prohibitive. The significant changes in conductance that take place during the redox transformation of an ICP recommend a closer look at the range of electrode potentials where these transformations occur for a comparison with the respective potentials of redox transitions of the inorganic constituent. As discussed previously [90], it might be important to determine whether the ICP keeps its highly conductive state during the redox transformation of the inorganic material or whether it stays in its neutral, poorly conductive state. The former case, preferable from the conductance point of view, lacks the Faradaic charge storage capability of the ICP just associated with this redox transformation. Even worse is an electrode potential excursion into the region of overoxidation of the ICP (for a discussion, see [91]). It is possible that conducting additives might be needed, and consequently, the ICP may or may not be able to perform some of the functions assigned to it above. Surprisingly, this aspect is almost never addressed in research reports. CVs of the composite constituents are not reported for such comparison, and neither are the in situ conductance data provided for the employed ICP or the composite obtained from electrochemically relevant electrode potentials; instead, standard amounts of conducting materials (and binder), with 15 wt.% being the most common case, are added.

ICPs have been proposed as active masses, first of primary and secondary batteries, and later of supercapacitors, since the discovery of their reversible redox behavior, suggesting employment of the proceeding transitions for charge storage [52,53,56,57,92,93]. Possible charge densities are large enough to suggest further investigations. Table 1 provides some typical data; both the calculated values and the experimental numbers may vary substantially from report to report, in most cases without apparent reason. Special care should be exercised when theoretical values exceeding even the most optimistic assumptions of the electrochemical reactions during charge storage are reported [94]; for a particularly noteworthy example, see [95]. In this example the egregious capacitance of 3407 F·g^−1^ is obviously due to a highly vague calculation of the electrode mass. In any case, this confirms concerns regarding the measurement and reporting of charge and capacitance data [39]. Possibly a similar case of a misquotation is the unspecified nanoporous material for a supercapacitor electrode with a surface area of 41.000 m^2^·g^−1^ as reported in [96]. 

The already addressed challenge caused by the variable electronic conductance of the ICP has been noticed early, and various attempts to ameliorate it have been made. In addition to the trivial addition of conducting materials, structuring at the micro- and nano level has been proposed; for an overview of the role of nanomaterials for supercapacitor electrodes with Faradaic storage, see [60,98,99,100,101,102]. The formation of, e.g., arrays of nanofibers, nanowires, or nanotubes, has been studied; for an overview, see [103]. Combination of, e.g., PANI with silica yielding a material with more exposed PANI surface has been suggested as another approach [104]. The markedly increased surface area enables high currents with low overpotentials. Unfortunately, the thin wires show significant potential drop along the wire. On the other hand, they also allow for better material utilization because of the better ordering without large particles [67,105]. Electrospinning may provide another approach to fibrous materials [106,107,108,109,110,111,112]. A more general overview on nanostructured ICPs and their structure-related advantages has been provided in [113]. In most cases, studies of the use of ICPs in supercapacitor electrodes start by addressing nanostructuring as an option to ameliorate the insufficient stability. Another approach at the focus of this report starts with the option of using composites, as was done, for example, in [58] with PANI as the ICP. Both approaches—structure-related and materials-related—are hard to separate, because they both end up at the same samples and electrodes. In any case, both the performance and the stability critically depend on the choice of the electrolyte and the electrolyte solution, as reviewed in [114].

A further drawback of most ICPs is their limited processability. They cannot be melted, and even more unfortunately, they are insoluble in most solvents (despite claims to the contrary in many reports apparently based on misunderstanding or the quoting of erroneous sources). Oligomers of some ICPs show limited solubility in some solvents, while other ICPs like PEDOT can be handled as dispersion. The latter case, also named solution-processable, actually does not refer to a true solution. Sometimes these advantages come at a price, in particular lower electronic conductivity of the product, or, quite obviously, difficult handling implications regarding the formation of their composites. A recent update on soluble but nevertheless highly conducting PANI and its composites shows some options [115]. These options include the use of specific counterions needed for the charge-balancing of the ICP in its oxidized, i.e., conducting, form.

In a review on the performance of ICP-based supercapacitors much beyond a simple recording of the storage capabilities, specific attention has been paid to the stability of materials and devices and the reasons for the sometimes poor long-term behavior and further general device properties [116].

Using graphene paper as a support and current collector has been called a composite in a somewhat unusual interpretation of the meaning of this term [4]. Options for improving the stability of ICPs in supercapacitor applications (leading straight to the composites discussed in Section 3.). Suitable structuring (arrays of fibers, wires, or columns) for both increased surface area and better accommodation of the volume change and formation of composites have been tried [4]. PANI nanostructures obtained by electroless (i.e., chemical oxidation) deposition were discussed in [117]; stability was not examined. An overview of the progress with plain ICPs as electrode material is available in [118].

The use of mathematical models for understanding materials’ properties and performance as well as in rational design development has been highlighted in [119], for reasons presumably beyond the scope of this contributions, not much has been reported on this direction.

## 4. The Combinations

Materials are briefly sketched in the following sections. Structural and other features assumed to be relevant for material performance are noted. Nonspecific claims like “highly suitable porosity” or “very efficient molecular interaction between the constituents” stated without any evidence are not repeated here. The storage capacity data for the material and/or an electrode made of it or a device are not repeated here. Without a commonly accepted measurement procedure and a standard of reporting results [39,120,121,122] these data will most likely be misleading. Due to the continuing discussion of the issue of whether the electrochemical response of a material should be designated as capacitive, pseudocapacitive, or battery-electrode-like (see above, and more on this below; the discussion borders on a religious debate sometimes [71]) and the related proposal to report the storage capability of materials acting like (double-layer) capacitor materials in Farad F (=As∙V^−1^), and the storage capability of battery-electrode-like materials in some other units like As (which apparently does not make sense, because it requires a well-defined electrode reaction equation describing a complete conversion of the electrode material, indeed assuming a specific number of transferred electrons per reaction as expected for a battery electrode but hardly for a supercapacitor electrode) with a specified electrode potential difference Δ*E* also yielding units As∙V^−1^ (which is indeed and expectedly the equivalent of F), terms and units as provided by the quoted authors (mostly capacitance) are used.

As mentioned above a major hindrance keeping new materials from consideration for practical application is deficient stability, whether only presumed or actually observed. Accordingly, stability data are quoted—even though they are disappointing sometimes at the current state of development. These data should be inspected cautiously.

The term cycle specifies mostly GCD-cycles; seldomly are cyclic voltammograms meant. The results of both types of cycle for the material are rather similar, and accordingly, no further distinction is made. The electrode potential window for every experiment is arbitrarily set by the authors. Its selection may affect the observed capacitance/charge. A wider electrode potential window frequently yields larger specific values, because the possibly higher material utilization is combined with the onset of parasitic reactions (overoxidation [91] and/or, in aqueous electrolyte solutions, hydrogen evolution at the negative electrode or oxygen evolution at the positive). This may result in diminished stability, particularly at the positive electrode due to the onset of degradation. Accordingly, the provided stabilities should be accepted with precaution.

In addition to the various possible functions performed by the constituents in a composite material, the motivations for studying and hopefully applying composite materials according to published research are:

- Enhanced stability (includes mitigation of volume changes, inhibition of materials dissolution)
- Enhanced conductivity

Improved conductivity as a general additional benefit has been noticed in an early review on ICPs and their composites in supercapacitors [9]. Enhanced material utilization enabled by suitable structuring balancing stability and utilization has been addressed in [58].

In an overview of ICP-based composites, nano- and microstructuring have been identified as key issues for optimized performance [61].

The following organization follows the identity of the other constituent and the number of constituents. The organization suggested in [18] and not even used by the presenting authors is not applied, as organizing composites following categories like “reinforcement” or “matrix” hardly appears to be practical or useful.

### 4.1. ICPs and Carbonaceous Materials

During early investigations, insufficient stability of ICPs as electrode materials first in secondary batteries and later also in supercapacitors, was already noted (despite frequently stated but unsupported claims to the contrary). Unfortunately, the stability of ICPs during cycling was not examined sufficiently in many of the reported studies. Insufficient stability was attributed to the significant volume change during cycling with the associated ingress/egress of counter-anions needed for charge balancing causing mechanical degradation [4,9]. The addition of carbonaceous material with a suitable shape (fibers, nanotubes, and nanowires) was suggested as an option to mitigate the undesired effects of the volume change; for early examples, see [123,124,125]. The quite obvious further benefits of enhanced and electrode potential-independent conductivity supporting higher electrode currents and a better electrode potential homogeneity inside the electrode were not explicitly addressed; they can nevertheless be assumed. This suggestion is tentatively supported by experimental evidence reported in [123]: the addition of acetylene black with a particulate morphology resulted in lower charge densities than with CNTs. The preparation of a composite material by plain mechanical mixing yielded an inferior material, obviously intimate mixing of both components—the already-formed nanotubes or AB particles and the polymer formed by chemical or electrochemical oxidation—is a prerequisite for the preparation of a promising material. The better performance obtained with CNTs instead of AB was attributed to “CNTs providing a better support,” though possibly “better dispersion” is a more adequate description [123]. Further support for this line of argument can be found in [126]; the electrodeposition of PANI in the presence of multiwalled carbon nanotubes MWCNTs in the deposition solution yielded a composite film of MWCNTs coated with PANI showing a lower internal resistance and a significantly greater specific capacitance. Better performance of a CNT/PANI-composite included significantly increased stability [11,127]. CNTs coated with PANI showed a fourfold charge storage capability as compared to plain CNTs [128]. Calling the carbonaceous component in a composite “filler” may be misleading [76,118]. The incorporation of CNTs as reinforcing nanowires has been reported with a reference to the use of these materials in supercapacitors in [129]. Combining solution—processable PANI:CSA (camphorsulfonic acid)—with CNTs yielded a flexible electrode and 98% capacity retention after 13,000 cycles [114]. Composites of CNTs with PPy have been reviewed [90,130]. Stability or capacity retention (if that is meant by “cyclability” in [130]) varies widely. A best value of 95% retention after 10,000 cycles in a device with a gel electrolyte was reported in [131]. Numerous applications of CNT/ICP composites, including those in supercapacitors, have been reviewed in [132,133]. The influence of the length of CNTs has been studied, with longer CNTs presenting a better overall performance and enhanced stability [134].

Single-walled carbon nanotubes SWCNTs have been coated electrochemically with PANI [135]. During subsequent electrodegradation (i.e., electrode potential cycling in a specified range of electrode potentials), a significant increase in capacitance was observed, which was attributed to the removal of only loosely attached fragments and to a growth of polycrystalline PANI regions.

After an early comparison of MWCNTs coated with various ICPs [136] and of promising results obtained with aligned MWCNTs coated with PPy [137] as supercapacitor electrodes, these composites with ICPs (as also with other second constituents) were reviewed [26,27]; the higher stability of the composites as compared to the plain ICP was highlighted. The particularly suitable pore size distribution (many mesopores) as compared to simple activated carbons, which enabled easy electrolyte solution and ion access, was stated as an advantage. Accelerated ion and electron transport evidenced from, e.g., electrochemical impedance measurements was identified as a major improvement for PPy coated on MWCNTs in comparison to the plain ICP [138]. Although no equivalent circuit was provided for the evaluation of the impedance measurements, it appears that the large low-frequency capacitance corresponds to the circuit element assigned elsewhere to the redox process in the ICP [39,139].

Carbon cloth or carbon paper and the corresponding graphitized materials appear to be suitable substrates; they may even be considered as constituents in composites. As opposed to the title of [140], carbon cloth is only one of several carbonaceous materials in this report; only two examples of composites with ICPs are mentioned. PANI nanowires formed on carbon cloth yielded a flexible electrode with 86% capacitance retention after 2100 cycles [141]; arrays of α-Fe_2_O_3_ on carbon cloth coated with PPy were studied as electrode in an asymmetric solid-state supercapacitor with 97% capacity retention after 5000 cycles [142]. Earlier, a similar approach with Ti-doped Fe_2_O_3_-nanorods on carbon cloth coated with PEDOT yielded an electrode with 96% capacity retention after 30,000 cycles [143].

Graphene (GN) in its various types and forms is already an attractive material for supercapacitor electrodes of the EDLC type [144], and particular attention has been paid to thermally expanded graphene for this application in [145]. The application of electrophoretic deposition in supercapacitor electrodes has been inspected in [146]. In addition, graphene oxide (GO) and reduced graphene oxide (rGO) (for unknown reasons rGO is called chemically converted graphene CCG [147]) have been studied for supercapacitor electrode applications. An early review of composites of CCG and ICP is available in [147].

The effects of preparation details on the synergistic effects between PANI and graphene in their composites have been discussed in [148]. The higher stability of the composite in comparison to that of plain PANI attributed to the presence of graphene did not yet show practically relevant results in the collected data. As a conclusion, careful optimization of all of the process parameters was stressed, and the introduction of a third constituent (see below Section 4.5) was proposed. A layered composite of graphene oxide and PPy was prepared and studied in [149]. A 70% capacity retention after 1000 cycles is certainly an improvement when compared with 30% for plain PPy, but it is still far away from practical relevance. Further reports on PPy/graphene composites have been surveyed [78], and the observed capacity retention vs. cycle number data could not match those found with PPy/CNT (see above). It is possible that the more likely electronic percolation with CNTs may be the crucial advantage; this requires further investigation and once more recommends optimization of the carbonaceous constituent fraction.

Further increase of the storage capabilities, and possibly also improved handling of graphene, by forming composites with ICPs has been suggested; some examples have been presented in [76,150,151,152,153,154,155,156]. Along with its composites, 3D-graphene has been highlighted in [157]; self-assembled 3D graphene macrostructures have been examined for supercapacitor application in [158]. For a 3D PANI/reduced graphene oxide composite, 94% capacity retention after 10,000 cycles were reported [159].

Composites based on 3D-graphene monoliths and their superior properties have been presented; beyond general claims, no stability data were included [160]. Particular advantages of 3D-architecture obtained with graphene/ICP-composites were highlighted in [161]. Synthetic methods of observed morphologies and performance data were presented without identifying a particular one as the most promising. Further improvements to stability were demanded without providing a critical look, at least at the state of the art. Graphene/ICP hydrogel composites have been studied in [162]. Although some materials showed noteworthy capacity retention during cycling, the authors stated the water content (freezing and evaporation) as a potential source of stability limitation. They left the real meaning of this concern open.

Nanocomposites of graphene with PANI [163,164,165,166,167,168] and with PTh have been reviewed in [169]. Oxidative chemical and electrochemical preparation were compared. Electropolymerization yielded deposits faster, though unfortunately mostly as thin films with irregular structure as compared to the more-or-less developed structures obtained by chemical polymerization. Numerous different materials and morphologies have been reported, and the determined storage capabilities range widely, though unfortunately not supported by stability data. Composites of graphene with various ICPs and with redox-active organic materials have been reviewed in [170]; more on the latter aspect can be found in [53]. The high electronic conductivity of graphene helps with charge delocalization in the material [170] and subsequently with enhancing material stability, presumably by avoiding uneven electrode potential distribution inside the electrode causing, e.g., overoxidation. Sometimes the interaction (presumably the strength of attachment up to covalent functionalization) has been found to be insufficient, thus negatively affecting both the performance and stability.

Possibilities of using the essentially 2D-surface of graphene (particles) as platform to attach ICPs have been highlighted in [151]. It appears that graphene/ICP composites need further research and optimization in terms of better attachment of the polymer to graphene with a suitable porosity supporting fast ion movement and thus high current capability [171].

Graphene inks possible useful in printed electronics, and related applications, including inks made of ICP-containing composites, have been reviewed in [172].

The nitrogen-doping of CNTs and graphene as a way to modulate the electronic and surface properties of the materials has been examined in [173]. With respect to their use in supercapacitor electrode materials, enhanced wetting resulting in better material utilization and the creation of surface functionalities by *N*-doping enabling redox charge storage or enhanced interactions with other redox-storage materials like PANI were observed.

An overview on the use of “nanocarbons” [174,175] and an update on carbonaceous materials [176] for supercapacitors are available.

Results for various composites of ICPs with graphene for use in micro-supercapacitors have been collected in [177], and the combination of reduced graphene oxide with PEDOT/PSS showed promising stability.

Further overviews and reviews dealing with composites of PANI with various carbonaceous materials are available in [58,59,178,179,180]. A capacity retention of 96% after 10,000 cycles was reported in [159].

A few further examples of composites of graphene and CNT with some ICPs can be found in [181]. Flexible supercapacitor sheets with embedded nanomaterials (e.g., CNT, but TiO_2_ particles and graphene flakes were also mentioned) have been prepared and tested, but nothing was stated regarding stability [182]. Very few composites with PPy have been surveyed [183]. A total of 99% capacity retention after 5000 cycles was found with a carbon/PPy composite in [184].

The various drawbacks and limitations of PIND have been described [185], though even composites with various carbonaceous materials showed hardly promising performance; at best, 91% after 5000 cycles was observed [186]. Further materials with PIND have been reviewed in [151].

Composites of PANI with quantum dots of various carbonaceous materials have been reviewed, but very few examples of tests as supercapacitor electrodes were mentioned [187]; for further examples, see [188,189,190]. In the latter case, the gravimetric storage capability of the composite increased by a factor of five at the optimum composition (10 wt.% quantum dots), 80% of the initial capacitance were left after 3000 cycles. Surface amino-functionalized nitrogen-doped quantum dots combined with PANI yielded a composite with significantly increased storage capability as compared to plain PANI depending on the quantum dot fraction [191]; unfortunately, the capacity retention was poor, with major drops after a few thousand cycles. Sulfur- and nitrogen-doped graphene quantum dots combined with PANI yielded a composite tested for supercapacitor electrode suitability. The addition of the quantum dots yields increased capacitance; stability was not examined [190]. Further examples of composites of ICPs with various carbonaceous quantum dots can be found in [192]. In a review on polymer dots, the author apparently confused polymers and metal oxides; the application of such polymer dots in supercapacitors has apparently not been reported so far [193].

Combinations of ICPs with biopolymers (e.g., lignin) opening the way to the utilization of materials from renewable sources have been examined [194,195,196].

Pyrolysis of biomass may also yield carbonaceous materials with large nitrogen contents favorable for supercapacitor applications, including composite formation [197,198,199,200]; (why such material is called an electrocatalyst in [201] in this application remains mysterious, the article is also confusing elsewhere).

### 4.2. ICPs and Chalcogenides

Beyond single-metal oxides (e.g., MnO_2_) [201,202,203,204,205,206,207,208,209,210,211], binary [212] and even ternary metal chalcogenides have attracted researchers’ attention as storage materials in supercapacitor electrodes because of their mostly rich redox chemistry with numerous possible redox transitions resulting in a more or less pronounced pseudocapacitive response [71].

The insufficient supercapacitor electrode performance of plain metal oxides was stated in [7,213]; it has been attributed to the frequently poor electronic conductance and solubility of the compounds, at least in certain redox states of the active metal ions resulting in fast capacity fading. As a remedy, nanostructuring has been proposed and examined [63,202,214,215,216,217]; the preparation of composites, including those with ICPs, has been recommended elsewhere [5,7,71,213,214,218,219,220]. To call such composite-formation a modification may be misleading [5]. Numerous composites of ICPs with metal chalcogenides with tailored microstructure optimizing electron and ion transport have been reviewed [61,71,214]. MnO_2_, as presumably the most popular metal oxide in supercapacitor research (and a bridge material in the ongoing merger between batteries and supercapacitors), has been coated with various ICPs [136]. Combination with these ICPs resulted in increased storage capabilities attributed in part to changed morphologies which in turn enable better material utilization. Stabilities were not addressed. For more examples, see [221] (this article is somewhat different from the title which suggests only carbon materials, as composite constituent ICPs are also treated).

In an overview on transition metal chalcogenides, mostly sulfides (in particular MoS_2_) have been collected with wildly varying capacity retentions ranging from 98% after 500 cycles to 98% after 8500 cycles [5]. More on composites of ICPs and MoS_2_ can be found in [222], again with highly varying capacity retentions from 90% after 500 cycles to 86% after 16,000 cycles.

Although Fe_3_O_4_ appears to be an attractive active mass, results obtained with its composites with ICPs are rare; this appears to be related to the poor electronic conductivity of the metal oxide (which is apparently not sufficiently enhanced by the ICP) and low stability [223]. Synthesis and characterization of various iron oxides of different dimensionalities have been reviewed with attention being paid to possible use in supercapacitor electrodes [224]. As opposed to the previously mentioned report, the high electronic conductivity of Fe_3_O_4_ is claimed as an advantage. Elsewhere in this report, low conductivity is deplored somewhat inconsistently. Promising results in terms of both capacity and stability were obtained with a core–shell type Ti-doped Fe_3_O_4_ coated with PEDOT [143].

V_2_O_5_ has been claimed to be a particularly suitable material, in particular when incorporated in composites, in [225]. Although the title of this contribution limits the scope to carbon-based composites, examples with ICPs are extensively discussed. Capacitance retentions around 90% after 5000 cycles were recorded as state-of-the-art, for practical applications, and further significant improvements may be necessary. In a report on the application of ruthenium oxide-carbon-based nanofiller-reinforced conducting polymer nanocomposites, PANI shows up only in the glossary [226]; a composite of RuO_x_ and PPy reported before in [227] was not examined for stability. Due to its large theoretical storage capability, CoO has attracted attention; very few of its composites were mentioned in [228], and stability data was not provided. Even less is known about the very few composites with copper oxides [229] and copper sulfides [230]. Conversely, there are numerous studies on Co_3_O_4_ and its composites, as reviewed in [231]. A core–shell array composite of Co_3_O_4_ and PANI showed 98% capacity retention after 5000 cycles in a complete flexible device with a gel electrolyte; this retention was observed after 10,000 cycles [232].

Further overviews and reviews dealing with composites of PANI with various metal oxides are available in [58,59]. In addition, some composites of ICPs with various rare earth metal oxides [233] and a few metal oxide/polyindole composites have been examined [78]. Contradictorily to the title (suggesting titanium/polymer composites), mostly TiO_2_-polymer composites were inspected as supercapacitor electrode materials, MXene’s with titanium appear in passing, in [234]. The stabilities of electrodes and/or devices with ICP-containing composites were not addressed.

As carbonaceous materials by themselves (except for a few redox functionalities provided by surface groups) do not add storage capability beyond double-layer storage, the obtained materials presented above show improved performance in terms of higher rate capability and better stability, but not necessarily higher storage capability. It should be noted nevertheless that sometimes substantial improvements were noticed (in comparison to a plain ICP) because of increased mass utilization. The large surface area of fibrous composite materials and the favorable morphology of the obtained electrodes have been highlighted in their review as a particular advantage [235]. Electrospinning has been addressed as a preparation method above [106].

### 4.3. Further Binary Combinations

Metal pyrophosphates of divalent metal cations (M) of the generic type M_2_P_2_O_7_ may undergo a redox reaction possibly suitable for charge storage.

M_2_P_2_O_7_ + 2 OH^−^ ⇆ M_2_P_2_O_7_(OH)_2_ + 2 e^−^ (1)

An overview is available in [236]. A composite of Mn_2_P_2_O_7_/PPy showed 99% capacitance retention after 10,000 cycles (without PPy, only 92%) in a complete device with an alkaline gel electrolyte [237], and the respective copper compound in Cu_2_P_2_O_7_/PPy yielded 99% after 6000 cycles (without PPy 86%) [238]. In both cases the composite had almost double the charge storage capability when PPy was present.

Metal–organic frameworks (MOFs) and their composites have been considered as active masses in supercapacitor electrodes [239,240,241,242]; combined with ICPs, the obtained composites were examined for various electrochemical applications [243]. The poor electronic conductivity of MOFs was noticed as a major hindrance to their use in a supercapacitor electrode; forming composites [156,244,245,246], including ICPs, is an obvious solution [77,242]. Only a few experimental observations focused on material science aspects were recorded. MOF/PANI composites were specifically discussed in [247,248]. Remarkable attention was paid to stability; capacitance retentions up to 86% after 2000 cycles were found.

Combinations of two ICPs by, e.g., coating PANI on an array of PPy-nanotubes [249] have been studied; for an overview see [58,59]. Such approaches should be distinguished from the use of copolymers [53,250].

In an otherwise hard to understand overview of metal-based supercapacitor electrode materials, ICPs were mentioned only in passing and were hardly relevant in recent context [251]. A composite of MXene/PEDOT:PSS has been reported [252] with remarkable AC-response performance. Further combinations of MXene’s with ICPs suggested in a somewhat hard-to-read overview [253] turned out to be misunderstandings. A composite of Ti_3_C_2_/PPy showed 83% capacity retention after 4000 cycles [254], overviews on MXenes in supercapacitors are available [255,256]. In a further overview of the nanocomposites of ICPs with MXene’s [257] again very few applications in supercapacitors were mentioned.

In a review of SiO_2_-based composite electrodes [258], ICP-coated silicon nanowires were described as electrode materials in hybrid micro-supercapacitors [259,260,261]. Contradictory to the suggestion provided by the title in [259], the silicon nanotrees in [260,261,262] were grown by chemical vapor deposition, not by the reduction of SiO_2_. PPy [262] and PEDOT [260] were subsequently electrodeposited. The silicon substrate provided some double-layer charge storage; the ICP contributed redox storage. After 10,000 cycles, 70% of the initial capacitance were retained with PPy; the PEDOT-coated silicon nanotrees retained 80% after 3500 cycles. The charge storage (not the energy storage as erroneously claimed by the authors in the title) of the Si/PEDOT composite has been studied with an electrochemical quartz crystal microbalance [262]. Both cations and anions of the used ionic liquid participated.

Nanocomposites of PANI with metal nanoparticles have been critically reviewed in [263]. The incorporation of gold NPs increased electronic conductance, beyond enthusiastic claims for large capacities, stability apparently has not been addressed. A Pt/PANI composite prepared by the electropolymerization of aniline in the presence of dispersed platinum particles in the deposition solution has been suggested for use in supercapacitor electrodes in [264]. The claimed improved porosity and increased electronic conductance could not be verified with the displayed data; a missing comparison with a plain PANI-electrode prevented any appreciation of the advantage, possibly caused by the added noble metal. A platinum-particle decorated PANI-film has been examined with respect to its capacitive properties in [265].

### 4.4. Ternary Composites with ICPs

The properties and performance of some of the binary composites inspected so far may be further improved by adding a third constituent (binder, conducting additives, and current collector once again are not counted); for an early approach with dismally poor stability data, see [266]. In a rational approach maintaining the various functions of constituents indicated above (sect. 1), the addition of even a small fraction of CNTs (enough to provide continuous pathways for electronic conductance, i.e., above the percolation level, which is easier to reach with one-dimensional objects [267]) may increase material utilization [39,40]. Apparently, a similar effect was achieved using silver deposited on PANI and subsequently coated with PANI and finally with MnO_2_ [268]. The direct exposure of the metal oxide to the organic electrolyte solution may be the cause of a capacitance retention of only 83% after 700 cycles. Adding metal oxide particles of a suitable shape (morphology) to a binary composite of ICP and carbonaceous compound may ensure a suitable and stable pore structure. This approach is nicely illustrated in a review [269] moving from PEDOT to PEDOT and carbon or MnO_2_ to PEDOT+carbon+MnO_2_, though unfortunately without providing any rationale behind it. Reviews of further material combinations are available [151]. An overview on MnO_2_-based ternary composites is available in [270]. Adding a carbonaceous component to MnO_2_/ICP composites generally increased stability. Capacity retentions > 90% after 1000 cycles were observed; 129% after 20,000 cycles appears to be an outlier.

Graphene as the carbonaceous component with ICPs and further constituents has been inspected [271,272]; 3D graphene oxide has been combined with CNTs and PANI into a freestanding paper-like electrode [273]. The good performance was ascribed to the highly conductive support of the carbonaceous constituents; stability was not examined. Graphene nanoplatelets have been combined with PANI and MoO_3_ into a ternary composite whose capacitance retention of 92% after 1000 cycles was claimed to be outstanding but is actually far from being of practical impact [274]. Further examples can be found in [275]. A macroporous ternary composite of PPy, graphitic C_3_N_4_, and graphene showed high electronic conductivity, high thermal stability, and 80% capacity retention after 2000 cycles [276]. A capacitance retention of 96% after 10,000 cycles was reported for a ternary composite of PANI, MnO_2_ and reduced graphene oxide [277].

A ternary composite of functionalized CNTs with MnO_2_ and polyindole showing 92% capacitance retention after 5000 cycles was prepared and tested in [278]. Possibly the “higher interactive behavior” suggested as an attractive feature of polyindole contributed to this stability. For further ternary composites with PIND, see [78].

The use of ICPs as a coating on metal oxide nanoparticles deposited on graphene has been proposed as an option to prevent the detachment of these particles during the operation of the electrode in an aqueous electrolyte solution [150]. Thin coatings were found to be sufficient and to be advantageous with respect to the utilization of the metal oxide. Presumably, these coatings also prevented the dissolution of the metal oxide in the electrolyte solution.

Further examples of ternary material combinations can be found in [58]. A focus on such combinations of ICPs, graphene, and metal oxides can be found in [279]. An array of TiN nanowires formed on carbon cloth was first coated with carbon, then with PANI [280]. A resultant 98% capacitance retention was found after 2000 cycles.

Invoking the obvious argument that deficits of one constituent can be made up by another, further constituent filling this deficit of quaternary composites has been reviewed [151]. A nanocomposite of CuO, NiO, PANI, and MWCNT was reported as an example [281]. Its remarkable gravimetric charge density, being a multiple of the corresponding ternary composite materials, was attributed to a very low apparent charge transfer resistance, possibly corresponding to the larger BET surface area and the larger pore volume, whereas the stability was attributed to the PANI-coating.

A ternary material containing lignin, PEDOT, and poly-aminoanthraquinone has been tested as electrode material and in an asymmetric supercapacitor in [196]. A significant effect of the deposition sequence (PEDOT/lignin coated with PAAQ or PAAQ coated with PEDOT/lignin) was noticed and explained in terms of the material properties of the layers). The former combination showed a better performance, especially in terms of improved stability.

Electrochemically inactive constituents may be added in order to establish and maintain a particularly suitable morphology of the electrode material, a specific porosity, large surface area, or flexibility. The incorporation of nanocellulose into composites of carbonaceous materials and an ICP apparently did not yield any significant advantage [282,283]. A fibrous matrix of functionalized CNTs and cellulose coated by chemical polymerization with PPy demonstrated a significant benefit of the added CNTs in terms of capacity retention after 5000 cycles from 68 to 110% [284]. To benefits of added CNTs in terms of increased conductivity and better performance concluded by the author, enhanced stability may be added, as already indicated above.

A composite of PANI, silver, and cellulose nanofibrils was prepared and tested in a flexible solid-state supercapacitor in [285]. As cellulose is an electric insulator (as stressed once more in [286] when reviewing the dismally poor capacitance data obtained with composites of various types of cellulose with ICPs) it was first processed into a hydrogel and subsequently decorated with silver particles. For increased storage capability, further PANI was deposited. Stability tests were not reported. Overviews on the use of bacterial cellulose in energy storage applications including supercapacitors are available [286,287].

The incorporation of nanoclay to a composite of PANI and CNTs obviously increased the electronic conductance of the material because of additional doping afforded by the clay [288]. A resultant 92% capacitance retention after 2000 cycles was reported; the actual mode of operation of the added nanoclay appears to need some further study.

### 4.5. Further Combinations

Graphene oxide/gold and reduced graphene oxide/gold nanoparticles have been coated/capped with ICPs with rather fuzzy conclusions regarding the role of the constituents and the obtained improvement (not talking about costs!) [289]. The advantages of nitrogen as part of an electrode material, in particular of carbonaceous materials in terms of improved performance, electronic conductance, etc., has been reviewed with a focus on polymeric carbon nitride in [290].

### 4.6. Miscellaneous Observations

Processes from biotechnology may be adapted to the formation of specific advantageous morphologies of ICPs and of their composites, as addressed in the preceding sections; for examples and a review, see [291]. Most ICPs of interest in electrochemistry and in electrochemical energy technology contain heteroatoms like N, S, or O. When they are pyrolyzed, these heteroatoms may stay in the obtained carbonaceous materials, providing them with enhanced electronic conductance and higher electrochemical activity. In addition, a particularly suitable morphology or architecture may be maintained [58,59,292]. A large nitrogen content in graphitic materials obtained from ICPs was found also to be advantageous in terms of storage capability [11]. Similar considerations with further chemical elements involved apply to MOF transformed into pyrolysis products (mostly metal oxides) or other materials [241,293,294,295,296]. In another (non-pyrolytic) procedure, a self-assembled *N*-doped carbon framework was assembled which was subsequently coated with PANI [197]. After 5000 cycles, the framework without PANI maintained 110% of the initial capacitance, whereas the PANI-coated material kept 80% of the initial value—twice that of the one without coating.

After praising the numerous advantages of polyindole as compared to polypyrrole, the rather disappointing outcome of a comparison between the performance data from both ICPs, attributed to the irregular backbone of the former ICP, is slightly surprising.

Composites of ICPs with the various other constituents discussed above have been studied as materials for flexible and paper-based supercapacitors [11]. In a review of materials for self-healing devices, supercapacitors with this capability have been treated [297].

The electrochromic performance of supercapacitors in functionalized devices has been reviewed [243]. Transparent supercapacitors (TSC or TCS, with C obviously representing conducting—the authors could not make up their mind about terminology) have been reviewed in [298]. A composite or reduced graphene oxide (apparently considered to be a metal oxide) with PANI was mentioned. The color changes of an organometallic compound, which was used as a charging indicator layer and for charge storage, combined with a CNT/PEDOT composite electrode yielded an electrochromic supercapacitor [299]. The device survived 1000 cycles without known capacity retention.

Progress of fiber-reinforced multifunctional composites for structural supercapacitors has been reviewed in [300]. Stability data pertaining to ICP-containing composites have not been reported so far.

Electrochemical techniques suitable for the preparation of micro-/nanostructured supercapacitor materials have been described in detail [301]. Examples of the simultaneous (one-step) deposition of metal oxide and ICP as well as two-step procedures are discussed. Layer-by-layer assembly (care should be exercised when comparing with multilayer assemblies which apparently are more likely to be composed of the same material) has been reviewed as an advantageous method to fabricate composites [302]. Given the procedural complications, the reported moderate capacity retentions possibly do not justify the extra effort. The 3D-printing of supercapacitors as a flexible approach to the production of energy storage devices has been studied [303].

## 5. Conclusions

Supercapacitor electrodes with only one kind of material (not counting auxiliary materials like binder and support) may show limitations in terms of storage and high current capability or stability. Combination with one or even two further active materials yielding a binary or ternary composite has been demonstrated as a promising option. Although frequently not even mentioned the beneficial effects of the added material can be deduced from the reported evidence in many cases. The selection of original reports, overviews, updates, and reviews evaluated in this report provides new insights into a deeper understanding of the observed benefits and improvements. Thus, it might offer inspiration for the rational selection of constituents and combinations yielding new and better performing materials. Beyond selecting and combining materials’ electrode architecture, optimized porosity and rational design carefully considering the functions of the various constituents must be included. Electrodes should be prepared with mass loadings and thicknesses closer to practical values. For practical application, long-term stability way beyond the frequently measured 1000 or 2000 cycles is mandatory; in future research more attention must be paid to this aspect. Another aspect is the inclusion of renewable materials of natural origin and possibly the exclusion of materials having very limited resources or being burdened with environmental or even health risks.

## Figures and Tables

**Table 1:** Some electrochemical data of the selected electrode materials ^1^.

Material	Molecular Weight of Repeat Unit/g	Oxidation Level */–	Theor. *Q* ^#^	Measur. *Q*/F·g^−1^
PANI	93	0.5	750 F·g^−1^ (Δ*E* = 0.7 V)	240
PPy	67	0.33	620 F·g^−1^ (Δ*E* = 0.8 V)	530
PTh	84	0.33	485 F·g^−1^ (Δ*E* = 0.8 V)	–
PEDOT	142	0.33	210 F·g^−1^ (Δ*E* = 1.2 V)	92
Quinone/HQ	108	2	1787 As·g^−1^	–
Ferrocene	185	1	522 As·g^−1^	–
Li	6.939	1	13,904 As·g^−1^	–
Al	26.98	3	10,728 As·g^−1^	–
PbO_2_	239	2	807 As·g^−1^	–

^1^ Data are mostly from [97]; there are numerous further sources with poorly specified data (mostly secondary ones) of unspecified origin, see, e.g., [77,78]. PANI = polyaniline, PPy = polypyrrole [also poly(2,5-pyrrolylene)], PTh = Polythiophene [also poly(2,5-thienylene)], PEDOT = poly-3,4-ethylenedioxythiophene. * Oxidation level, also “dopant level”, specifies the fraction of oxidized repeat units in the polymer, as well as the number of electrons formally transferred in the electrode reaction. ^#^ Gravimetric charge density can be stated with respect to the electrode reaction in units As·g^−1^, or in the case of a material where no clear electrode reactions can be stated, as the amount of charge stored within a change of electrode potential Δ*E* in units of As·V^−1^·g^−1^ (i.e., F·g^−1^).

## Data Availability

Not applicable.
